# The content of African diets is adequate to achieve optimal efficacy with fixed-dose artemether-lumefantrine: a review of the evidence

**DOI:** 10.1186/1475-2875-7-244

**Published:** 2008-11-25

**Authors:** Zulfiqarali G Premji, Salim Abdulla, Bernhards Ogutu, Alice Ndong, Catherine O Falade, Issaka Sagara, Nathan Mulure, Obiyo Nwaiwu, Gilbert Kokwaro

**Affiliations:** 1 Department of Parasitology/Medical Entomology, School of Public Health and Social Sciences, Muhimbili University College of Health Sciences, Box 65011, Dar-es-Salaam, Tanzania; 2 Ifakara Health Research and Development Centre, Dar-es-Salaam, Tanzania; 3 Centre for Clinical Research, Kenya Medical Research Institute, Kisumu, Kenya; 4 Centre for Nutrition Education and Research, Nairobi, Kenya; 5 Clinical Pharmacology Department, University College Hospital, Ibadan, Nigeria; 6 Malaria Research and Training Center, University of Bamako, Bamako, Mali; 7 Novartis Pharma AG, Nairobi, Kenya; 8 Novartis Pharma AG, Lagos, Nigeria; 9 Kenya Medical Research Institute (KEMRI)/Wellcome Trust Programme, Nairobi; 10 Department of Pharmaceutics and Pharmacy Practice, College of Health Science, University of Nairobi, Kenya

## Abstract

A fixed-dose combination of artemether-lumefantrine (AL, Coartem^®^) has shown high efficacy, good tolerability and cost-effectiveness in adults and children with uncomplicated malaria caused by *Plasmodium falciparum*. Lumefantrine bioavailability is enhanced by food, particularly fat.

As the fat content of sub-Saharan African meals is approximately a third that of Western countries, it raises the question of whether fat consumption by African patients is sufficient for good efficacy. Data from healthy volunteers have indicated that drinking 36 mL soya milk (containing only 1.2 g of fat) results in 90% of the lumefantrine absorption obtained with 500 mL milk (16 g fat). African diets are typically based on a carbohydrate staple (starchy root vegetables, fruit [plantain] or cereals) supplemented by soups, relishes and sauces derived from vegetables, pulses, nuts or fish. The most important sources of dietary fat in African countries are oil crops (e.g. peanuts, soya beans) and cooking oils as red palm, peanut, coconut and sesame oils. Total fat intake in the majority of subSaharan countries is estimated to be in the range 30–60 g/person/day across the whole population (average 43 g/person/day). Breast-feeding of infants up to two years of age is standard, with one study estimating a fat intake of 15–30 g fat/day from breast milk up to the age of 18 months. Weaning foods typically contain low levels of fat, and the transition from breast milk to complete weaning is associated with a marked reduction in dietary fat. Nevertheless, fat intake >10 g/day has been reported in young children post-weaning. A randomized trial in Uganda reported no difference in the efficacy of AL between patients receiving supervised meals with a fixed fat content (~23 g fat) or taking AL unsupervised, suggesting that fat intake at home was sufficient for optimal efficacy. Moreover, randomized trials in African children aged 5–59 months have shown similar high cure rates to those observed in older populations, indicating that food consumption is adequate post-weaning. In conclusion, it appears that only a very small amount of dietary fat is necessary to ensure optimal efficacy with AL and that the fat content of standard meals or breast milk in sub-Saharan Africa is adequate.

## Background

Progress in the ontrol and treatment of malaria in Africa has been threatened by growing resistance to previously effective therapies, notably chloroquine and sulphadoxine/pyrimethamine. Combination therapy is now widely regarded as the most effective strategy for limiting the emergence and spread of resistance and combination treatment based on artemisinin is now recommended by the World Health Organization (WHO) [1].

Artemether-lumefantrine (AL, Coartem^®^, Novartis Pharma AG, Basel, Switzerland) combines the artemisinin-derivative artemether, which has a prompt onset of action and provides fast symptomatic relief, with lumefantrine, which has a slower onset of action and clears residual parasites [2]. AL has consistently shown high efficacy and tolerability in African adults and children with uncomplicated malaria caused by *Plasmodium falciparum*, achieving 28-day polymerase chain reaction (PCR)-corrected parasitological cure rates of >95% [3-7]. AL is the first and only fixed-dose artemisinin-based combination treatment (ACT) pre-qualified by the WHO, and has now been adopted as first-line treatment for uncomplicated malaria due to *P falciparum *in more than 20 African countries including Kenya, Malawi, Uganda, Tanzania, Mozambique, Nigeria, South Africa and Ethiopia. Many other countries have also switched to AL as first-line therapy. The manufacturer introduced price reductions in 2006 and 2008, cutting the public sector price by approximately 50% since 2001.

Cost-effectiveness assessments, one based on data from a randomized trial in Tanzania [8] and another based on public health data collected in the field in Zambia [9], have confirmed that AL offers a cost-effective treatment option compared to other antimalarial drugs.

Artemether and lumefantrine are lipophilic molecules. Food, especially dietary fat, enhances the bioavailability of both agents, but the effect is more pronounced with lumefantrine [2]. The fat content of African meals tends to be relatively low; indeed, total fat consumption in sub-Saharan Africa is approximately a third that of Western countries [10]. This raises the important question of whether adults or children prescribed AL in sub-Saharan Africa consume sufficient fat to ensure parasite clearance. This article reviews the typical components and fat content of African diets and consider the adequacy of fat intake in terms of achieving optimal efficacy.

### Characteristics of lumefantrine absorption

Lumefantrine, like several other lipophilic antimalarial agents, is absorbed slowly after oral administration, and bioavailability varies between individuals [2,11-13]. Concomitant food increases lumefantrine bioavailability [14]. In a double-blind trial of 260 patients with uncomplicated malaria in Thailand, Ezzet *et al *showed that both the extent and variability of lumefantrine absorption improved with clinical recovery as normal food intake was resumed [14]. Oral bioavailability increased by 108% when a normal meal was eaten close to AL dosing, compared to the fasting condition. Adequate lumefantrine absorption is important in order to avoid low plasma concentrations with risk of treatment failure [2].

**Figure 1:**
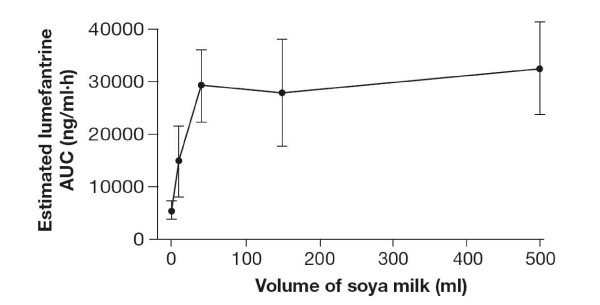
Relationship between volume of soya milk consumed and estimated lumefantrine absorption (AUC) in healthy volunteers. (Reprinted with permission ) [15].

The key question for the healthcare team is how much dietary fat is necessary to achieve complete parasite clearance with AL. This question has been investigated by Ashley *et al *in a cross-over pharmacokinetic study in which lumefantrine exposure (area under the curve, AUC) was measured in healthy volunteers after a single dose of AL was administered with different volumes of soya milk or with no milk [15]. Based on these data, the authors constructed a population model which indicated that in this population of healthy volunteers, drinking 36 mL of soya milk (containing only 1.2 g of fat) resulted in 90% of the lumefantrine exposure obtained with 500 mL milk (16 g fat); 74 ml of soya milk (2.4 g of fat) achieved 99% of the exposure seen with 500 mL (Figure 1). Ashley and colleagues pointed out that 1.2 g of fat can be obtained in a remarkably small amount of food: for example, a single teaspoon of vegetable oil contains 5 g of fat.

### Typical components of African diets

Diets in Africa vary widely according to regional, economic, environmental and cultural factors. Nevertheless, there are certain typical patterns of food consumption (Table 1). African diets are traditionally based on a carbohydrate staple that is supplemented by soups, relishes and sauces derived from a wide range of other foodstuffs. The carbohydrate component may be primarily cereals (e.g. maize, sorghum, millet, rice), starchy roots (e.g. potatoes, sweet potatoes, yams, cassava) or fruit (plantain), depending on the country and local area. In recent years there has been a trend away from the consumption of roots towards maize, rice and wheat, particularly in cities [16]. Accompaniments to the carbohydrate staple are usually composed of vegetables (e.g. eggplant, cabbage, carrots, spinach, kales, French beans, onions), pulses, nuts (particularly peanuts), meat (chicken, beef or pork) or fish, depending on local availability, household income, season and community habits, with the greatest variety seen in coastal regions and the fertile highlands [16].

**Table 1:** Typical components of regional African diets for adults and infants

**Region**	**Adults**	**Infants**
East Africa	Starchy foods (e.g. cooked bananas, potatoes, cassava, corn, wheat products, rice). Food consumed to accompany starches includes beef, lamb, poultry, stews made of legumes, soups, vegetables (mostly fried in cooking oil or fat or cream) and peanut/groundnut-based sauces	Exclusively 4–6 months unless contraindicated. Weaning may start from month 3 onwards Supplementary milk from cows Early food: porridge containing milk or coconut oil.
West Africa	Bean balls fried with oil, maize fortified with palm oil, groundnuts, bread, eggs, rice, yam, beef or fish stew, yams, vegetables (mostly fried in cooking oil)	Exclusively breast-fed up to 4 to 6 months unless contraindicated Supplementary milk (cow milk or baby formula) Early foods include chocolate drinks, mashed yam, beans
Central Africa	Rice, cassava, sorghum, millet, vegetables (fried in peanut oil), palm oil, fish, meat	Exclusively breast-fed up to 6 months Supplementary milk from cows Early food: porridge from maize meal and milk
Southern Africa	Maize, mashed potatoes, vegetables, beef, rice, bread, cassava	Exclusively breast-fed up to 6 months unless contraindicated Supplementary milk from cows Early food: porridge maize meal

The Food and Agriculture Organization (FAO) of the United Nations compiles data on various aspects of food production and availability [17]. These statistics offer a valuable source of information, although it should be borne in mind that estimates are based on the amount of food available for human consumption, rather than actual amounts eaten, and do not account for wastage. Table 2 summarizes the consumption of selected food groups in sub-Saharan countries based on FAO data, and highlight the predominance of cereals and starchy root vegetables [18].

**Table 2:** Average consumption of selected food groups in subSaharan African countries during 2001–2003 [18]

	**Average intake (g/person/day)**										
	**Cereals**	**Fish, seafood**	**Fruit**	**Meat**	**Milk**	Oilcrops*	**Pulses**	**Starchy roots**	**Sugars, sweeteners**	**Vegetable oil**	**Vegetables**
Burkina Faso	601	6	14	30	56	42	14	16	15	13	47
Burundi	84	5	284	10	13	2	103	632	8	2	93
Cameroon	288	38	213	41	40	19	39	400	28	21	217
Congo, Dem Republic of	102	16	82	13	4	12	10	842	8	15	25
Côte d'Ivoire	331	40	201	31	21	20	1	619	30	37	103
Ethiopia	384	1	28	23	61	2	35	168	12	4	31
Ghana	249	80	319	27	21	35	2	1107	19	16	88
Kenya	337	12	154	40	263	4	42	164	55	20	102
Malawi	403	10	107	14	12	13	34	504	31	9	54
Mali	502	21	8	51	126	5	25	30	30	18	66
Mozambique	289	6	48	15	12	5	26	665	31	19	17
Nigeria	399	20	185	23	20	21	26	593	31	38	167
Sudan	370	5	83	59	410	11	22	13	55	21	135
Uganda	170	20	556	31	67	35	65	561	24	5	55
United Republic of Tanzania	307	19	81	27	70	13	28	518	21	14	76
Zimbabwe	355	4	32	42	54	17	13	43	97	26	30

* Excluding products

The most important sources of dietary fat in African countries are oil crops (e.g. peanuts, soy beans) and oils obtained from vegetables or plants such as red palm oil, groundnut oil, coconut oil and sesame oil. These are widely used in food preparation, for frying or as additions to sauces and stews. Additional contributions to fat intake are made by other commonly-used foodstuffs such as nuts and pulses. Table 3 describes the typical fat content of some staple foodstuffs [19]. While food composition inevitably varies according to crop variety, growing conditions and freshness, these illustrate that relatively small amounts of certain widely-consumed items such as soya beans and ground nuts contain high levels of fat. A further contribution comes from whole-grain cereals if the cereal germ is not separated out prior to milling: for example, maize contains 4.8 g/100 g [19] and is a staple food in many countries.

**Table 3:** Fat content of typical foodstuffs [19]

**Type**	**Food**	**Fat content (g/100 g)**
Carbohydrates and legumes	Maize	4.8
	Millet	3.5
	Rice	0.5
	Sorghum	3.2
	Wheat flour	2.0
	Cassava meal	0.5
	Beans	1.5
	Lentil	1.2
	Pigeon peas	1.3
	Soya beans	18.0
	Yam	0.1
Meat/fish	Beef	18.0
	Chicken	6.5
	Eggs	10.0
	Fish	7.4
Nuts/fruit	Peanuts	45.0
	Coconut	35.0
	Sunflower	46.0
	Avocado	18.0

### Total fat intake in African countries

Total energy intake per person across sub-Saharan Africa is currently estimated to be 2220 kcal/day, compared to 3340 kcal/day in developed countries [10]. Regional differences exist, most notably a higher average energy intake in West Africa compared to other areas (Table 4). Estimates of total fat intake from the FAO for the period 2001–2003 suggest that the average total amount of fat consumed per person per day vary considerably between subSaharan African countries (Table 5) [10]. The majority of countries, however, fall into the range 30–60 g/person/day. The daily intake of fat across subSaharan Africa is estimated to be 43 g/person/day, with the highest fat intake in West Africa (Table 1); for comparison, people in developed countries consume 123 g/person/day, on average [10]. The per capita daily intake of fat has generally remained stable or shown an upward trend in the majority of African countries over the last three decades [10], with exceptions being due to major conflicts or famines.

**Table 4:** Average dietary energy and fat consumption in regions of Africa. [10]

**Region**	**Dietary energy consumption** **(kCal/person/day)**	**Dietary fat consumption** **(kCal/person/day)**
SubSaharan Africa	2220	43
Central Africa	1830	36
East Africa	2040	35
Southern Africa	2080	36
West Africa	2580	57

**Table 5:** Average dietary fat consumption in sub-Saharan African countries during 2001–2003 [10]

	**Total fat consumption (g/person/day)**
Burkina Faso	56
Burundi	10
Cameroon	46
Congo, Democratic Republic of	26
Côte d'Ivoire	59
Ethiopia	20
Ghana	38
Kenya	49
Malawi	33
Mali	46
Mozambique	33
Nigeria	63
Sudan	69
Uganda	32
United Republic of Tanzania	31
Zimbabwe	55

### Fat content of breast milk and weaning foods

It is standard to breast-feed infants up to 4–6 months in all regions of Africa, although supplementary cow's milk may be given. The fat content of breast milk in rural African areas varies with the season, stage of lactation and between individual mothers, but an investigation in a farming community in The Gambia recorded an average fat content of 40 g/L breast milk [20], similar to that reported in other parts of the world [21-23]. Breast-fed infants in The Gambia have been shown to receive 15–30 g fat/day from breast milk up to the age of 18 months, with breast milk continuing to make a major contribution to fat intake even after partial weaning [24]. Comparing infants in The Gambia and the United Kingdom, Prentice *et al *found that fat intake from breast milk was at least as high in the Gambian children while being breast-fed exclusively [24].

Infants usually continue to receive breast milk until two years of age, with some food introduced from three months or later. The first weaning food is usually porridge made from a cereal such as rice, corn or millet mixed with water, or possibly cow's milk, with salt and sugar. Such porridges have a relatively low fat content (e.g. 0.1–0.3 g/100 g); although addition of soya milk or peanuts in some regions increases the fat content (1.2–1.9 g/100 g) [25]. In West Africa there tends to be a greater diversity of early foodstuffs, including chocolate, mashed potatoes or beans (Table 1).

The transition from breast milk to weaning foods and then to adult foods is associated with a marked reduction in the proportion of energy that is obtained from fat. A study in Kenya was undertaken in infants during the second year of life (mean 14–20 months) to assess fat intake over the period of weaning [26]. In these young children, the mean quantity of breast milk ingested was calculated to be 502 ± 239 g per 24 hours, although this varied widely between individuals [26]. The authors calculated the average fat intake from milk to be approximately 17 g/day, which was supplemented by 10.5 g/day from weaning foods. In the period prior to weaning, fat intake from breast milk decreased to approximately 11 g/day but after complete weaning the increased food consumption provided only an additional 3.5 g/day. A further detailed study in The Gambia has shown that total fat intake remained relatively stable over the first 17 months of life, but as the percentage of other foods (notably cereals and peanuts) increased in the diet, the percentage of energy from fat decreased from 50% during breast-feeding to 30% during mixed feeding and to 15% after total weaning [24]. Nevertheless, total fat intake, which was ~25 g/day until complete weaning, was >10 g/day subsequently.

Cow's milk is only given to infants infrequently in many African countries because of the low milk yield per animal and scarcity of preservation equipment, although it is more common in some countries (e.g. Kenya, Somalia and Sudan). Where cow's milk is used, however, it is a good source of fat with a fat content of 30 g/L [19], only slightly lower than breast milk.

### Relevant experience from clinical trials

In most trials of AL in African populations, AL has been administered under supervision with food or drink given in the hospital or clinic [4,7,27-29]. Two studies, however, have assessed 'real-life effectiveness' of AL, in which the drug was taken unsupervised at home [6,30]. These are helpful in providing an indication of whether normal, unsupervised food consumption during AL administration ensures good efficacy. Piola *et al *undertook a randomized trial in 957 patients in Uganda to compare the effectiveness of AL when administered under supervision in hospital with fat intake ensured by a meal comprising ~300 mL milk (10 g fat) with 30 g peanuts (13 g fat) versus AL taken unsupervised at home [6]. In the unsupervised group, only the first dose was taken in the clinic, and patients were then discharged and advised to take subsequent doses with a meal that contained fat (with no fat content specified), or breast milk in breastfed infants. The 28-day PCR-corrected cure rate in the evaluable population was 100% in both cohorts (95% CI 98–100 in the supervised group and 99–100 in the unsupervised group). The type of meal eaten at home was not recorded, so it cannot be determined if patients ate especially fatty meals or standard food. Lumefantrine plasma concentration was measured on days 3 and 7 in a random selection of 433 patients taking part in the study [31], and multivariate modelling of these data confirmed that supervised treatment was significantly associated with higher lumefantrine concentrations (p < 0.0001) [31]. In another trial, undertaken in an area of Tanzania with a high level of resistance to sulfadoxine-pyrimethamine and amodiaquine, Mutabingwa and colleagues randomized 1292 children aged 4–59 months with uncomplicated malaria to AL or to conventional drugs [30]. All agents were taken at home, without supervision, and no specific advice was given regarding concomitant food consumption. At 28 days, 97.3% of patients receiving AL showed parasitological cure after adjustment for recrudescence.

Together, these findings suggest that food consumption during unsupervised AL dosing in African patients, including children, provides sufficient fat to ensure effective parasitological clearance.

As discussed above, the fat consumption of infants shortly after weaning can be relatively low. It is highly relevant, therefore, that randomized trials in African children aged from five months [32,33] or 12 months [34] up to 59 months have consistently shown 28-day PCR-corrected parasitological cure rates of greater than 95% i.e. similar to cure rates observed in older populations.

It should be noted that data are scarce concerning lumefantrine absorption, and the effect of food consumption, in patients with HIV/AIDs or other coinfections such as tuberculosis. Pharmacokinetic and pharmacodynamic studies are required in the HIV/AIDS population, particularly in the light of increased antimalarial failure rates in such patients due to increased parasite burden and reduced host immunity associated with HIV infection (1).

## Conclusion

Although lumefantrine bioavailability is enhanced by fat, pharmacokinetic evidence suggests that only a very small amount of dietary fat is required [15]. In the event of varying dietary fat intake, such as during unsupervised dosing where fat consumption was not controlled [6] or was not specifically advised [30], the efficacy of AL does not appear to have been impaired. This is consistent with published statistics concerning the dietary fat intake of African populations, which indicate that average fat consumption is generally in the range of 30–60 g/day in subSaharan African countries. Even in young children shortly after weaning, in whom fat intake tends to be low, it has been estimated that fat intake exceeds 10 g/day. In summary, there is good evidence to suggest that the content of standard African diets or breast milk is adequate to ensure excellent efficacy for AL.

## Abbreviations

ACT: artemisinin-based combination treatment; AL: artemether-lumefantrine; AUC: area under the curve; FAO: Food and Agriculture Organization; PCR: polymerase chain reaction.

## Competing interests

The authors declare that they have no competing interests other than COF, who received a research grant from Novartis in 2002, and NM and ON, who are employees of Novartis Pharma AG.

## Authors' contributions

All authors provided critical review of the text and approved the final version.
